# The potential associations of periapical lesions on endodontic microsurgery with guided bone regeneration: a retrospective analysis of 23 cases

**DOI:** 10.3389/fmed.2026.1901096

**Published:** 2026-08-21

**Authors:** Yusheng Meng, Xiuqiao Yang, Yun Liu, Shuang Wang, Dongming Zeng, Min Wu

**Affiliations:** 1 Stomatology Health Care Center, Shenzhen Maternity and Child Healthcare Hospital, Women and Children’s Medical Center, Southern Medical University, Shenzhen, Guangdong, China; 2 Shenzhen Clinical Research Center for Obstetrics & Gynecology and Reproductive System Diseases, Shenzhen, Guangdong, China

**Keywords:** endodontic microsurgery, guided bone regeneration, periapical lesion, preliminary associations, radiographic analysis

## Abstract

**Objective:**

This single-center, retrospective study analyzed the histological and radiographic changes of periapical lesions, evaluated healing outcomes after endodontic microsurgery combined with guided bone regeneration, and explored the preliminary factors associated with these outcomes.

**Methods:**

All records were reviewed to identify patients treated by EMS with GBR at the Shenzhen Maternity and Child Healthcare Hospital between 2022 and 2026. The periapical tissues were stained with hematoxylin and eosin for morphologic and histopathological analysis. Preoperative and follow-up radiographic changes were calculated using Mimics software according to the PENN 3D criteria for three-dimensional (3D) healing. Survival and their preliminary factors were analyzed using logistic regression, and odds ratios (ORs) and 95% confidence intervals (CIs) were calculated.

**Results:**

A total of 23 patients (23 teeth) met the criteria for radiographic periapical healing analysis and were included in the survival analysis at 12-month follow-up. The histopathological images primarily demonstrate seven cases of periapical granuloma and 16 cases of radicular cyst. 3D reconstruction in Mimics effectively visualized periapical radiographic changes by enhancing the intuitiveness and reliability of outcome analysis. The overall radiographic periapical healing rate was 78.26%, and it was significantly associated with the gray values of periapical lesions (*p*: 0.023; OR: 0.091; 95% CI: 0.011–0.717) and deep overbite/overjet parameters of malocclusion (*p*: 0.035; OR: 0.071; 95% CI: 0.006–0.834).

**Conclusion:**

The gray values and increased overbite/overjet parameters of malocclusion may be considered potential univariate associations in the preliminary analysis, as 3D reconstruction in Mimics effectively visualized periapical radiographic changes. Meanwhile long-term follow-up studies of additional cases are still required to confirm these conclusions.

## Introduction

Root canal therapy (RCT) remains an effective treatment for periapical diseases (1). However, studies indicate that approximately 20% of teeth with chronic periapical lesions cannot be completely cured through simple RCT alone (2). After RCT, affected teeth may still present clinical symptoms such as swelling, persistent occlusal pain, and non-healing sinus tracts. Radiographical images also show an expanded transmission range of periapical tissues (3). These clinical and imaging manifestations are attributed to extensive resorption of apical bone. The response of periapical tissues to bacterial infection triggers host cells to release various inflammatory mediators, pro-inflammatory cytokines, and growth factors through the innate and adaptive immune systems (4). Although the molecular mechanisms remain incompletely understood, studies have confirmed that inflammatory mediators play critical roles in the onset, progression, and expansion of periapical diseases during the pathogenesis of pulp and periapical lesions (5). For teeth with persistent disease after RCT, endodontic re-treatment may be considered. However, challenges often persist: factors such as apical overfilling, root canal calcification, post-core restoration, or severe external periapical infection can lead to refractory periapical periodontitis. To preserve these affected teeth, endodontic microsurgery (EMS) has become the preferred treatment option (6).

Compared with traditional EMS, modern EMS uses more advanced instruments. For example, the application of dental microscopes improves surgical visualization, enabling more precise operations (7). The use of ultrasonic apical preparation and microsurgical instruments makes the procedure more minimally invasive, and the introduction of biocompatible materials improves the outcomes of apical sealing and bone defect repair (8). Minimally invasive techniques such as mini bone fenestration and apical curettage are used to remove periapical pathological tissues, followed by root resection, inverted root canal preparation, and inverted filling to achieve tight apical sealing and promote the repair of periapical soft and hard tissues (9).

For periapical bone loss caused by extensive periapical inflammation, guided bone regeneration (GBR) can be applied to the regeneration of periapical tissues. The complex morphology of bone defects and insufficient bone wall support may impair osteogenesis in the apical region. Extensive bone loss in this area can lead to the apical defect being filled with fibrous connective tissue, resulting in pathological scar formation rather than real tissue regeneration, thus adversely affecting the long-term prognosis. Performing GBR during EMS may be optimal in terms of surgical timing. A recent meta-analysis including 11 studies showed that regenerative techniques with the adjunctive use of collagen membrane and hydroxyapatite significantly improved the outcomes of EMS (10). Surgical outcomes are evaluated through comprehensive clinical and imaging analysis with cone-beam computed tomography (CBCT) imaging technology, which is recommended as a superior tool for assessing healing after periapical surgery. Compared with periapical X-rays, CBCT can evaluate three-dimensional spatial changes through coronal, sagittal, and axial views and enable comprehensive healing assessment (11). Surgical outcomes are affected by multiple factors, including patient gender, age, occlusal relationship, oral hygiene habits, size of periapical lesions, pathological changes, tooth position, quality of root canal treatment, and intraoperative materials (12).

This study aims to analyze the histopathological and radiographic changes of periapical lesions in EMS with GBR and further evaluate the relationship between therapeutic efficacy, clinical pathology, imaging healing status, and comprehensive patient associations after EMS with GBR for periapical lesion management.

## Materials and methods

### Data preparation

**Figure 1:**
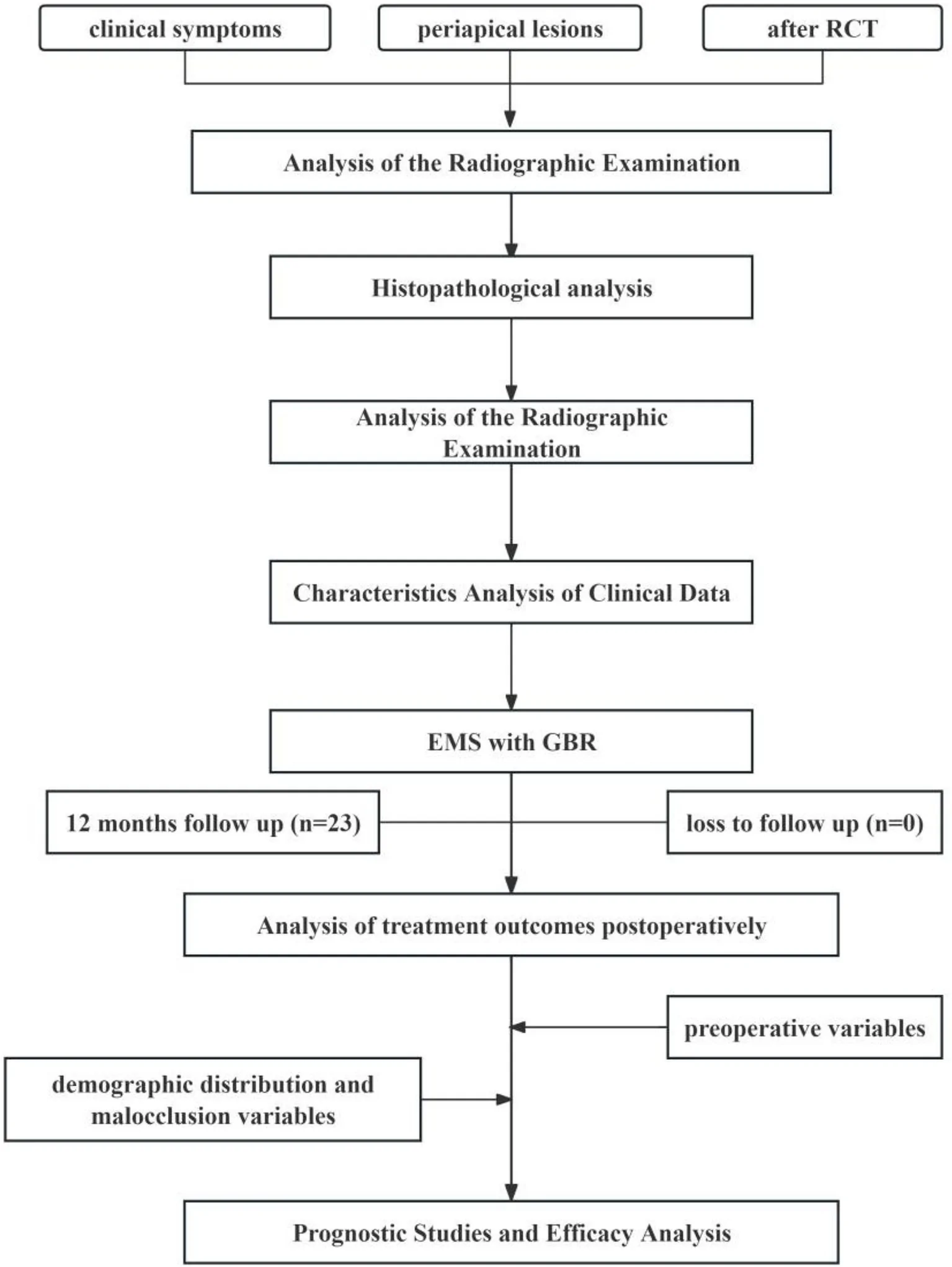
Flow chart of the study design and participants throughout the study.

This study was designed as a single-center retrospective study to evaluate patient- and tooth-related factors for EMS with GBR. We retrospectively analyzed patients who underwent EMS with GBR and completed a 12-month follow-up at the Department of Oral Surgery between May 2022 and January 2026. After obtaining approval from Shenzhen Maternity and Child Healthcare Hospital (approval No. SFYLS[2026]068), 23 teeth from 23 patients who received EMS with GBR were included in this study. All treatments were performed in accordance with standard surgical operating procedures. Patients were recalled for follow-up 12 months after RCT. Teeth with persistent periapical lesions defined as unsuccessful periapical healing on radiological imaging or with persistent discomfort in the affected tooth were indicated for EMS. The inclusion criteria for affected teeth were previous apical microsurgery of teeth that involved GBR; complete clinical data including medical history records, pathology examination results, and cone-beam computed tomography (CBCT) data; and a signed informed consent form from the patient for the procedure. The exclusion criterion were missing or incomplete clinical data. The flowchart of this retrospective study is shown in Figure 1. This study was conducted in accordance with the Helsinki Declaration of 1975, revised in 2013 by the World Medical Association.

### Surgical procedure

The patients had previously been treated before surgery, and the symptoms were recurrent. Patients used mouthwash containing 0.12% chlorhexidine gluconate (Peridex™) for 2 days before the surgery. The endodontic microsurgical procedures were performed by surgical operators according to the routine EMS protocol with Kim and Kratchman’s guidelines (13). Following a review of the patient’s medical history, the standard procedural steps for endodontic microsurgery were performed as follows: administration of sufficient local anesthesia using 4% articaine hydrochloride combined with 1:100,000 epinephrine, gingival incision, elevation of a full-thickness flap, osteotomy via a handpiece (manufactured by NSK, Japan), enucleation of the periapical cyst followed by irrigation with sterile saline, and staining of the resected root surface with methylene blue (produced by Jichuan, Taixing). If a root crack was found, the affected tooth was extracted. A small cotton ball containing epinephrine (YunDa, Wuhan) was used to stop bleeding. An ultrasonic treatment device (Satelec, France) and an ultrasonic retro-preparation tip (B&L Biotech, South Korea) were used to retro-prepare the root apex about 3 mm. iRoot BP Plus (BC, Canada) was used for retrofilling the root apex along the long axis of the tooth for more rigorous sealing of the apical region. The apex was resected at approximately 3 mm, ensuring the root cross-section was perpendicular to the long axis of the root. This depth typically eliminates most apical bifurcations and collateral canals. After mechanical hemostasis was achieved with an adrenaline-containing cotton ball, the resected apical section was stained with methylene blue solution to allow microscopic examination for fractures, microleaks, isthmus defects, or missed canals. The canal lumen was cleaned intermittently along its course to a depth of at least 3 mm using ultrasound. After drying the cleaned site, the canal was filled with iRoot BP Plus and compacted layerwise until dense sealing was achieved. Finally, the bone cavity was thoroughly irrigated with normal saline to check for any retained foreign bodies. The cases also involved guided bone regeneration (GBR) with Bio-Oss (Geistlich Pharma AG, Switzerland) and, depending on sinus involvement, application of Bio-Gide (Geistlich Pharma AG, Switzerland), accurate repositioning of the osteomembranous flap, and tension-free suturing. The surgical procedures were performed under a dental microscope (Mediworks, China) except for the incision, flap elevation, repositioning, and suturing. The periapical tissues were immediately fixed with 10% Neutral Buffered Formalin (NBF) and then submitted for histopathological examination. Patients were recommended to take ibuprofen (0.2–0.4 g) every 4–6 h to relieve pain, and antibiotics such as amoxicillin were administered when necessary. Sutures were removed 7 days after surgery, and all cases were followed up with clinical and radiographic assessments at 12 months.

### Tissue processing for morphologic and histopathological analysis

For morphological analysis, hematoxylin–eosin staining was performed on periapical tissue sections, following the experimental protocols outlined here. Immediately following extraction, the periapical tissue was placed in 10% buffered formalin solution for fixation and preservation. The tissue underwent a serial dehydration process using ethanol solutions of progressively increasing concentrations, followed by clearing with xylene. Subsequently, individual lesion tissue specimens were embedded in paraffin. The prepared paraffin-embedded blocks were sectioned into slices with a thickness of 5 micrometers. For the deparaffinization procedure, the 5-μm sections were first treated twice with xylene, then rehydrated through a gradient series of ethanol solutions with decreasing concentrations. Following rehydration, the sections were rinsed sequentially with tap water and distilled water over a 15-min period, then stained by immersion in hematoxylin solution for 3 min. After hematoxylin staining, the sections were rinsed with tap water for 15 min, transferred to 70% ethanol solution, and subsequently counterstained by immersion in eosin solution for 5 min. Following counterstaining, the specimens were subjected to four consecutive soaks in anhydrous ethanol and three consecutive soaks in xylene. Finally, the stained sections were mounted on glass slides with Entellan mounting medium. Microscopic examination of the hematoxylin–eosin-stained slides was conducted using a Nikon Eclipse E200 optical microscope (Nikon, Japan).

### Radiographic analysis

The CBCT images were obtained with the use of Planmeca Viso (Planmeca Viso G5, Finland) at a resolution of 150 microns. They were taken before the EMS (T0) and at the 12-month follow-up (T1). To maintain the consistency of measurement results, the CBCT clinical scanning protocol was fixed to a 10 × 10 cm field of view, 0.2 mm voxel size, 360° rotation, at 90 kVp, and the tube current modulation was used. CBCT images of patients with the preoperative and follow-up evaluation at T0 and T1 were imported into the Mimics software (Materialise, Belgium) in the DICOM format. A new project was created using DICOM files in Mimics. Firstly, the region-growing module was used to define the boundaries of the periapical lesion and the affected tooth; to obtain optimal visualization of the periapical lesion, we selected the primary view with the clearest margins and the three orthogonal plates, the anterior and posterior boundaries were set to the normal periapical bone of adjacent teeth, the superior boundary to the normal periapical bone surrounding the lesion, and the inferior boundary to the crown of the affected tooth. Next, a new mask was created. To ensure comparability across all cases, standardized window widths and grayscale thresholds were uniformly applied for semi-automatic segmentation, with threshold values determined by comparing lesion margins to the normal periapical bone of adjacent teeth. The segmentation thresholds were set to −700–1,000 for the periapical lesion, 1,000–3,071 for the tooth, and 226–3,071 for the periapical bone. The mixed mask editing tool was used to erase the normal bone surrounding the lesion, obtaining a preliminary lesion mask. The boundaries were then carefully inspected layer by layer on the sagittal, coronal, and axial plates. Finally, the “calculate 3D from mask” command was used to fill the lesion and construct the 3D lesion model, which was then optimized by removing any irregular surface spikes. The same method was used to obtain 3D reconstruction models of both the affected teeth and the bone graft areas. Subsequently, a three-dimensional reconstruction image was generated to calculate the lesion volume and gray values. To minimize variations caused by human factors, the same segmentation protocol and parameters were employed for both T0 and T1 CBCT scans. Identification of the lesion, tooth, and grafting area was performed with Layer-by-layer optimization by the axial, coronal, and sagittal plate, respectively. These procedures were calibrated and completed by three clinical radiologists with extensive experience in radiological analysis. Three radiologists analyzed the volume of apical lesions and GVs using Mimics software, employing a bivariate mixed model and performing multiple measurements on the same lesion area. If discrepancies among the three physicians exceeded a predefined threshold (>10%), all reviewers examined the images to resolve disagreements; once consensus was reached, measurements were repeated or a final value was determined. The final result was the average of all three measurements. The intraclass correlation coefficient (ICC) was calculated (typically accompanied by a 95% confidence interval), and lesion volume and GV data were included in the analysis only when ICC > 0.75.

### Assessment criteria

To assess the treatment outcome of EMS, clinical and radiographic records were analyzed preoperatively and at the 12-month follow-up after the surgical procedure. Clinical evaluation included assessment for any of the following symptoms and signs: spontaneous pain, percussion pain, swelling, tooth mobility, periodontal condition, and gingival sinus tracts.

Radiographic healing was qualitatively evaluated according to the modified PENN 3D criteria by Safi (14). Radiographic healing of the case was classified as complete, incomplete, uncertain, or unsatisfactory healing. The volume and GVs of periapical lesions were calculated by Mimics software from preoperative to the follow-up according to the studies (15, 16). Evaluation criteria for efficacy during the follow-up period.

Successful:

1) Clinical symptoms: no subjective discomfort, and clinical examination was asymptomatic.
2) Radiographic imaging results indicated complete healing: An intact lamina dura was restored across both resected and unresected root surfaces. Hard tissue completely covered the resected root end and full grafting material filling in the periapical lesion area; meanwhile, there was a visible lamina dura between adjacent bone tissue. The GVs of periapical lesions were adjacent to the apex and approached that of the surrounding unaffected bone tissue.

Failure (any of the following criteria indicating failure):

1) Subjective discomfort or clinical symptoms on examination, such as non-healed sinus tracts, gingival swelling, significant tenderness on percussion, apical tenderness upon palpation, and increasing mobility or worse periodontal condition.
2) Radiographic imaging revealed incomplete healing, uncertain healing, or non-healing: the cortical continuity was interrupted by a focal low-density area; the osteotomy site with grafting had not fully ossified; a low-density region remained adjacent to the resected root surface; or the low-density area was unchanged or larger in volume.

### The prognostic factors for the success rate of EMS between the preoperative and the follow-up periods

1. Patient gender and age, tooth group, primary etiology, and root canal treatment status.
2. The presence of symptoms (swelling or pain, sensitivity in response to percussion, and/or palpation).
3. Radiographic evaluation: Presence/absence of cortical bone destruction and the GVs of the periapical lesion.
4. Histopathological analysis: Pathological tissue analysis and morphological typing of the apical region.
5. The patient’s general condition: occupation, body weight, malocclusion, and oral hygiene.

### Statistical analysis

Statistical analysis was performed using software SPSS 26.0 (IBM SPSS Statistics 26). Qualitative data were analyzed using the *χ*^2^ test or Fisher’s exact probability method. The paired comparisons were performed using the Bonferroni adjustment method, with a two-sided significance level of *α* = 0.05. For quantitative data, the independent samples t-test or one-way ANOVA was employed, with a two-tailed significance level of *α* = 0.05. Logistic regression analysis was performed to assess the effect of age, gender, size of lesion, preoperative swelling, etiology, histology of lesion, tooth location, malocclusion, the GVs of periapical lesion, and cortical bone destruction, with a probability of *p* < 0.05 assigned as the level of significance, and multiple testing was performed. The ICC coefficient was required to be greater than 0.70 for acceptable agreement.

## Results

In total, 23 teeth from 23 individual patients were enrolled in this analysis. The 12-month postoperative follow-up results demonstrated that 18 teeth from 18 patients attained clinically satisfactory outcomes, whereas five teeth from the remaining five patients presented with suboptimal therapeutic effects. Based on the three-dimensional Penn criteria, the 12-month postoperative surgical success rate was calculated as 78.26%. The specific conditions of the cases with suboptimal outcomes were as follows: three teeth were extracted secondary to tooth or crown fracture; one tooth with preoperative periodontal-endodontic combined lesions exhibited no improvement in clinical manifestations; and one tooth developed bone graft infection accompanied by fistula formation 6 months after the surgical procedure.

### Radiographic and mimics analysis

**Figure 2:**
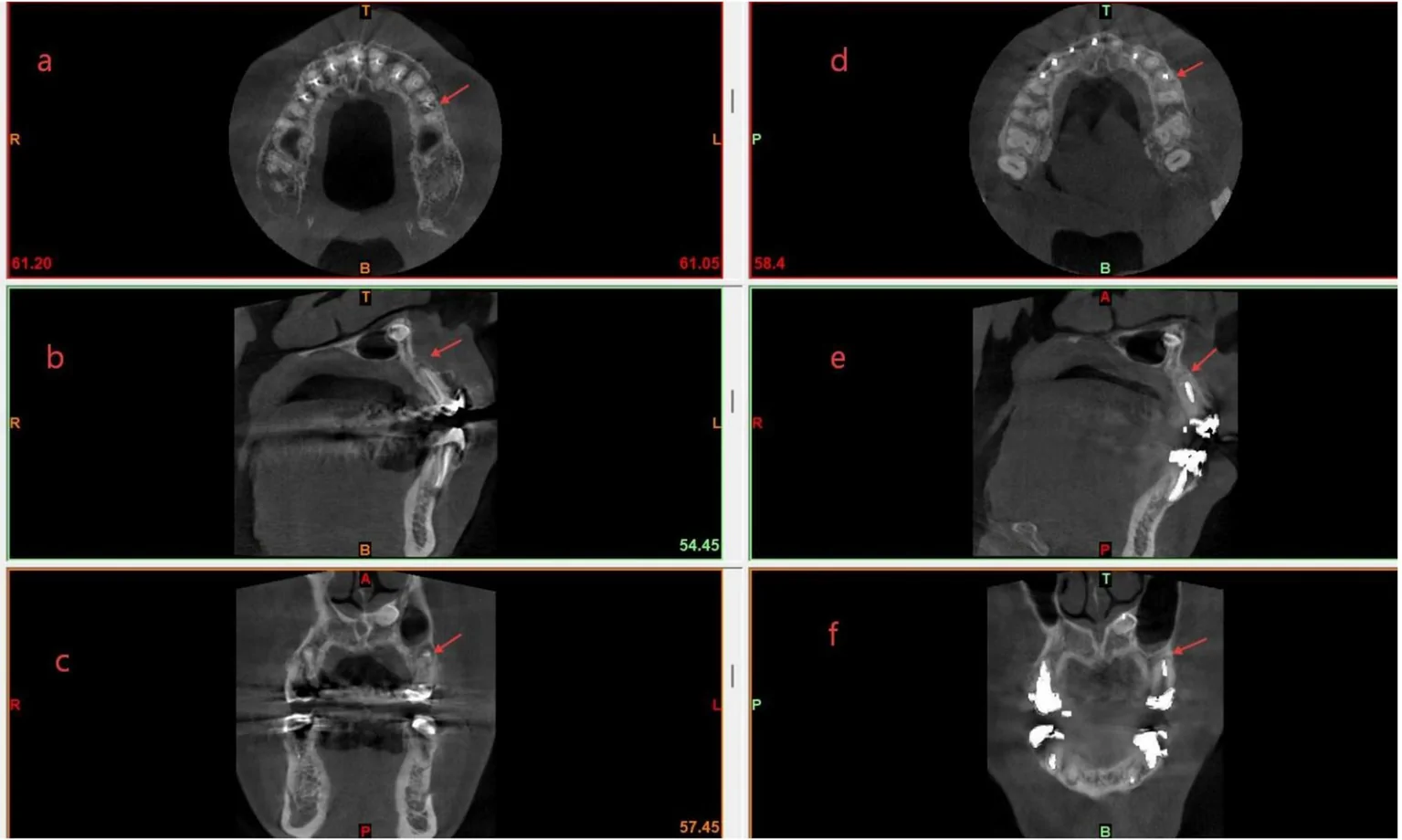
The red arrow indicates the lesioned area and the surgically healed area. CBCT images at T0 and T1 were imported into Mimics software and analyzed from three perspectives (in panels **a, d**; panels **b, e**; panels **c, f**). The three-dimensional imaging clearly demonstrates complete healing of the apical bone graft material and autologous bone tissue.

**Figure 3:**
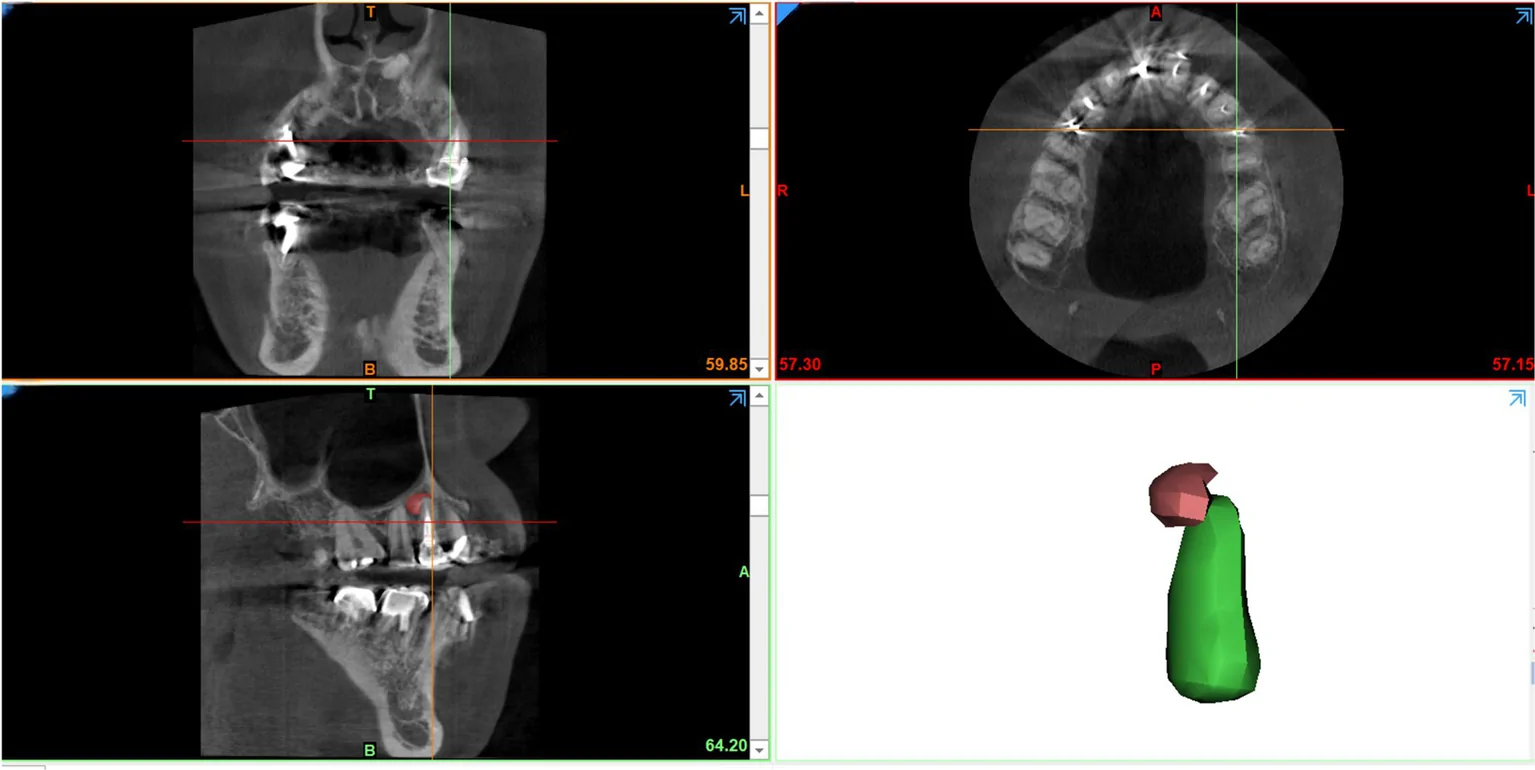
The calculation of lesion volume for a periapical lesion using Mimics software, 3D reconstruction showing the periapical lesion area (maroon) and periapical condition (green).

**Figure 4:**
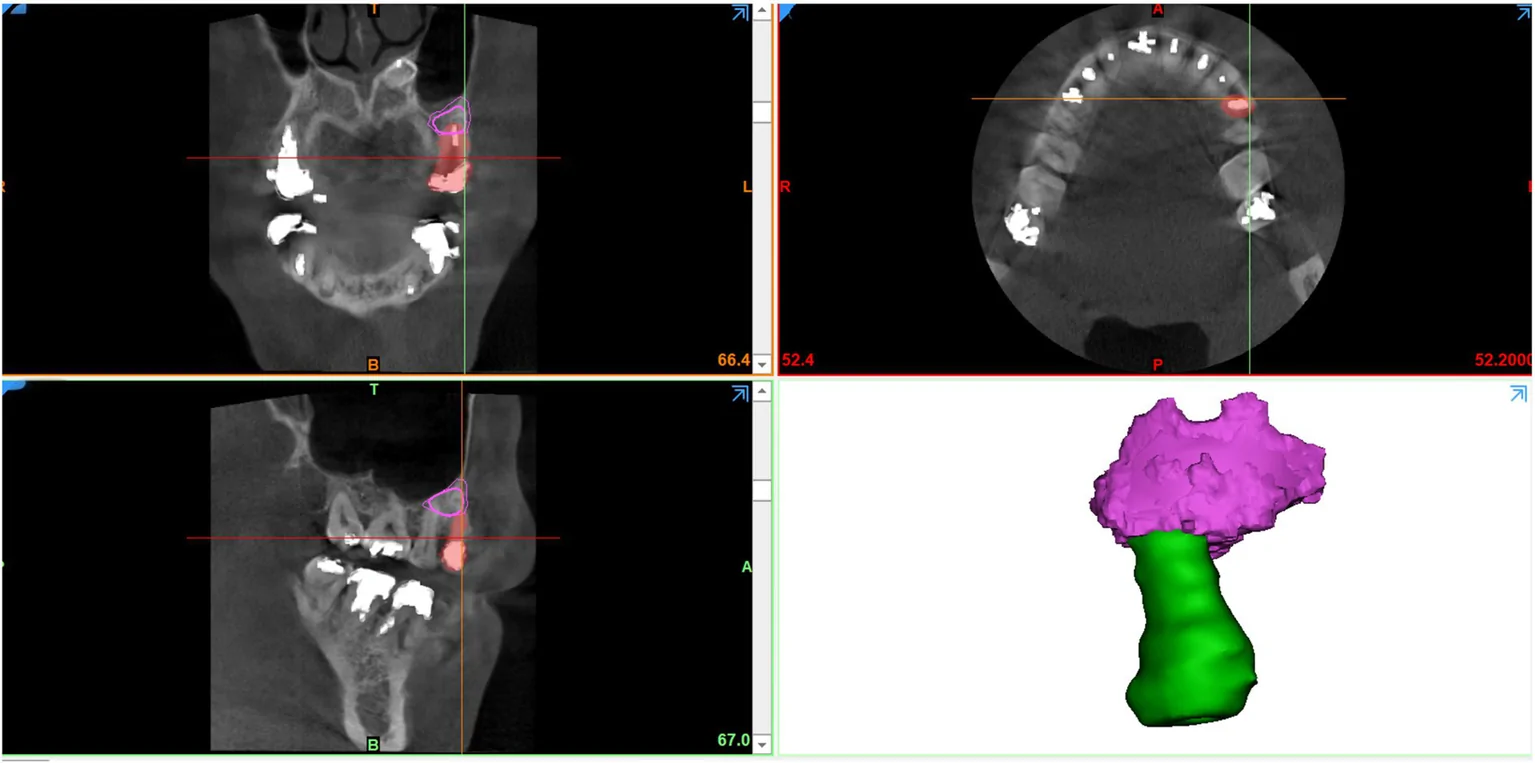
Complete healing of periapical lesion tissue with GBR; the hard tissue (purple) completely covered and encapsulated the resected apex (green) in 3D reconstruction by the Mimics.

CBCT images obtained at the EMS (T0) and 12-month follow-up (T1) were imported into Mimics software. The mean ICC for the quantitative variables was 0.98 (95% CI 0.994–0.999), which showed excellent agreement among three radiologists. Analysis was performed on the coronal, vertical, and horizontal planes, and complete healing of the apical bone graft material and autologous bone tissue was observed, as shown in Figure 2. The CBCT imaging data were imported into the Mimics software with the same voxel size and field of view settings for three-dimensional reconstruction of the periapical lesion areas. A region of interest (ROI) encompassing the periapical areas with GBR at 12-month follow-up and surrounding periapical periosteum and grafting material were defined for each case. Analysis of the grafting area (original lesion site) and the surrounding bone tissue gray-level threshold occurred at T1. Then, 3D reconstruction was performed to show the periapical lesion area, demonstrated in Figure 3. Analysis of the image grayscale values at T1 showed the full segmentation of the grafting material and the periapical bone, as their threshold values are close, as shown in Figure 4. 3D reconstruction and complete healing of periapical lesion tissue with GBR meant the hard tissue completely covered and encapsulated the apex.

### Histological analysis

**Figure 5:**
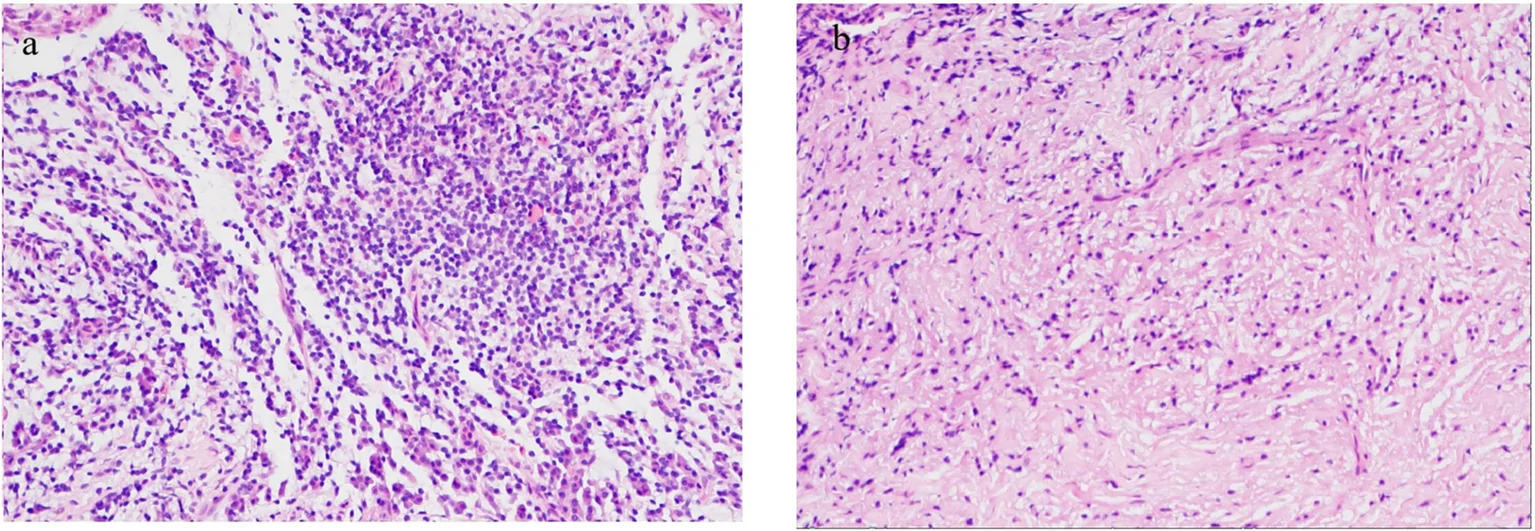
Periapical granuloma group **(a)**: Inflammatory cell infiltration within the periapical granulomas can be observed. These inflammatory cells include histiocytes, lymphocytes, and plasma cells. Additionally, numerous neutrophils, well-defined foamy macrophages, and vascular structures are visible within the pericapillary granulomas. Radicular cyst group **(b)**: The surface of the radicular cyst is covered with non-keratinized stratified squamous epithelium, with irregular hyperplasia of the epithelial papillae accompanied by extensive chronic inflammatory cell infiltration and intercellular edema. No significant inflammatory cell infiltration is observed in the fibrous layer, which is predominantly composed of collagen fibers. Cracks resulting from cholesterol crystal deposition are visible within the cyst wall tissue, often surrounded by a foreign body giant cell reaction (H & E; original magnification × 400).

The histopathological images primarily demonstrate seven cases of periapical granuloma (Figure 5a), showing inflammatory cell infiltration within the periapical granuloma, characterized by highly developed granulation tissue, prominent vascular structures, and extensive infiltration of lymphocytes, plasma cells, and histiocytes. Additionally, there were numerous neutrophils, well-defined foamy macrophages, and vascular structures. Figure 5b shows 16 cases of radicular cysts wherein the normal periodontal tissue structure is absent. The surface of the radicular cyst was covered with non-keratinized stratified squamous epithelium, with irregular hyperplasia of the epithelial pinnacles accompanied by extensive chronic inflammatory cell infiltration and intercellular edema. No significant inflammatory cell infiltration was observed in the fibrous layer, which is predominantly composed of collagen fibers. Cracks resulting from cholesterol crystal deposition were visible within the cyst wall tissue, often surrounded by a foreign body giant cell reaction.

### Logistic regression

In all, a total of 23 teeth/23 patients were entered into the analysis. The overall success rate was 78.26% (18 out of 23). Table 1 presents the distribution of cases according to tooth-related associations, radiographic parameters, preoperative-related variables, and histology of the lesion. None of the parameters in the tooth-related factors and histology of the lesion reached statistical significance; however, there was statistical significance in the variables of the GVs of periapical lesion (P: 0.023; OR: 0.091; 95% CI: 0.011–0.717). Table 2 showed the logistic regression model of demographic distribution and malocclusion variables, including body weight, blood glucose, oral hygiene, and malocclusion. Deep overbite/overjet of the malocclusion parameters had a significant association with EMS (P: 0.035; OR: 0.071; 95% CI: 0.006–0.834).

**Table 1:** Logistic regression model of preoperative variables.

Parameter		Total (*n*)	Success (completely healing) *n*(%)	Failure (uncertain, incomplete, or unsatisfactory healing) *n*(%)	OR (95% CI)	Logistic regression (*p* value Univariate)
Preoperative swelling	Present	11	8 (72.73%)	3 (27.27%)	0.533 (0.071–4.006)	0.541
	Absent	12	10 (83.33%)	2 (16.67%)		
Tooth location and type	Anterior	13	10 (76.92%)	3 (23.08%)	1.090 (0.276–4.304)	0.902
	Premolar	7	6 (85.71%)	1 (14.29%)		
	Molar	3	2 (66.67%)	1 (33.33%)		
Primary etiology	Injury	4	3 (75.00%)	1 (25.00%)	0.265 (0.049–1.447)	0.125
	Decay	12	9 (75.00%)	3 (25.00%)		
	Periodontitis	7	6 (85.71%)	1 (14.29%)		
Root canal treatment status	Initial root canal treatment	13	10 (76.92%)	3 (23.08%)	0.981 (0.268–3.595)	0.977
	Retreatment	6	5 (83.33%)	1 (16.67%)		
	No root canal treatment	4	3 (75.00%)	1 (25.00%)		
Apex diameter	<1 mm	11	8 (72.73%)	3 (27.27%)	0.981 (0.971–4.595)	0.973
	1 mm–2 mm	10	9 (90.00%)	1 (10.00%)		
	>2 mm	2	1 (50.00%)	1 (50.00%)		
Volume of a periapical lesion	<1,000 mm^3^	7	5 (71.43%)	2 (28.57%)	0.652 (0.177–2.405)	0.521
	1,000–1,500 mm^3^	9	7 (77.78%)	2 (22.22%)		
	>1,500 mm^3^	7	6 (85.71%)	1 (14.29%)		
The GVs of periapical lesions	<500	3	1 (33.33%)	2 (66.67%)	0.091 (0.011–0.717)	0.023*
	500–800	7	4 (57.14%)	3 (42.86%)		
	>800	13	13 (100%)	0 (0%)		
Cortical bone destruction	Palatal or buccal cortical plate	8	6 (75.00%)	2 (25.00%)	0.773 (0.098–5.405)	0.782
	Palatal and buccal cortical plate	15	12 (80.00%)	3 (20.00%)		
Periodontitis	Present	9	7 (77.78%)	2 (22.22%)	0.678 (0.107–7.487)	0.856
	Absent	14	11 (78.57%)	3 (21.43%)		
Histology of the lesion	Radicular cyst	16	13 (81.25%)	3 (18.75%)	1.733 (0.220–13.675)	0.602
	Periapical granuloma	7	5 (71.43%)	2 (28.57%)		

OR, odds ratio; CI, confidence interval; *n*, sample size; *show significance.

**Table 2:** Logistic regression model of demographic distribution and malocclusion variables.

Parameter		Total *n*	Success (complete healing)	Failure (uncertain, incomplete, or unsatisfactory healing)	OR (95% CI)	Logistic Regression (P Value Univariate)
Gender	Female	14	11 (78.57%)	3 (21.43%)	1.048 (0.138–7.934)	0.964
	Male	9	7 (77.78%)	2 (22.22%)		
Age	20–35 years	10	8 (80.00%)	2 (20.00%)	1.587 (0.338–7.465)	0.558
	36–50 years	11	9 (81.82%)	2 (18.18%)		
	>50 years	2	1 (50.00%)	1 (50.00%)		
Occupation	Time-fixed	15	12 (80.00%)	3 (20.00%)	1.333 (0.178–10.254)	0.782
	Time-free	8	6 (75.00%)	2 (25.00%)		
Body weight (BMI)	<18.5	3	2 (66.67%)	1 (33.33%)	0.687 (0.172–2.744)	0.595
	18.5–24	9	7 (77.78%)	2 (22.22%)		
	>24	11	9 (81.82%)	2 (18.18%)		
Blood glucose	Abnormal	6	4 (66.67%)	2 (33.33%)	0.492 (0.052–3.521)	0.431
	Normal	17	14 (82.35%)	3 (17.65%)		
Oral hygiene	Absent	5	4 (80.00%)	1 (20.00%)	0.785 (0.257–3.048)	0.846
	Mediocre	7	5 (71.43%)	2 (28.57%)		
	Optimal	11	9 (81.82%)	2 (18.18%)		
Smoking	Occasional	6	4 (66.67%)	2 (33.33%)	0.636 (0.201–2.012)	0.441
	Frequent	5	4 (80.00%)	1 (20.00%)		
	Never	12	10 (83.33%)	2 (16.67%)		
Malocclusion						
Crowding	Present	11	9 (81.82%)	2 (18.18%)	1.501 (0.201–11.236)	0.693
	Absent	12	9 (75.00%)	3 (25.00%)		
Deep overbite/overjet	Present	8	4 (50.00%)	4 (50.00%)	0.071 (0.006–0.834)	0.035*
	Absent	15	14 (93.33%)	1 (6.67%)		
Cross bite	Present	10	7 (70.00%)	3 (30.00%)	0.424(0.056–3.213)	0.407
	Absent	13	11 (84.62%)	2 (15.38%)		

OR, odds ratio; CI, confidence interval; *n*, sample size; *show significance.

### Typical case reports

#### Case 1

**Figure 6:**
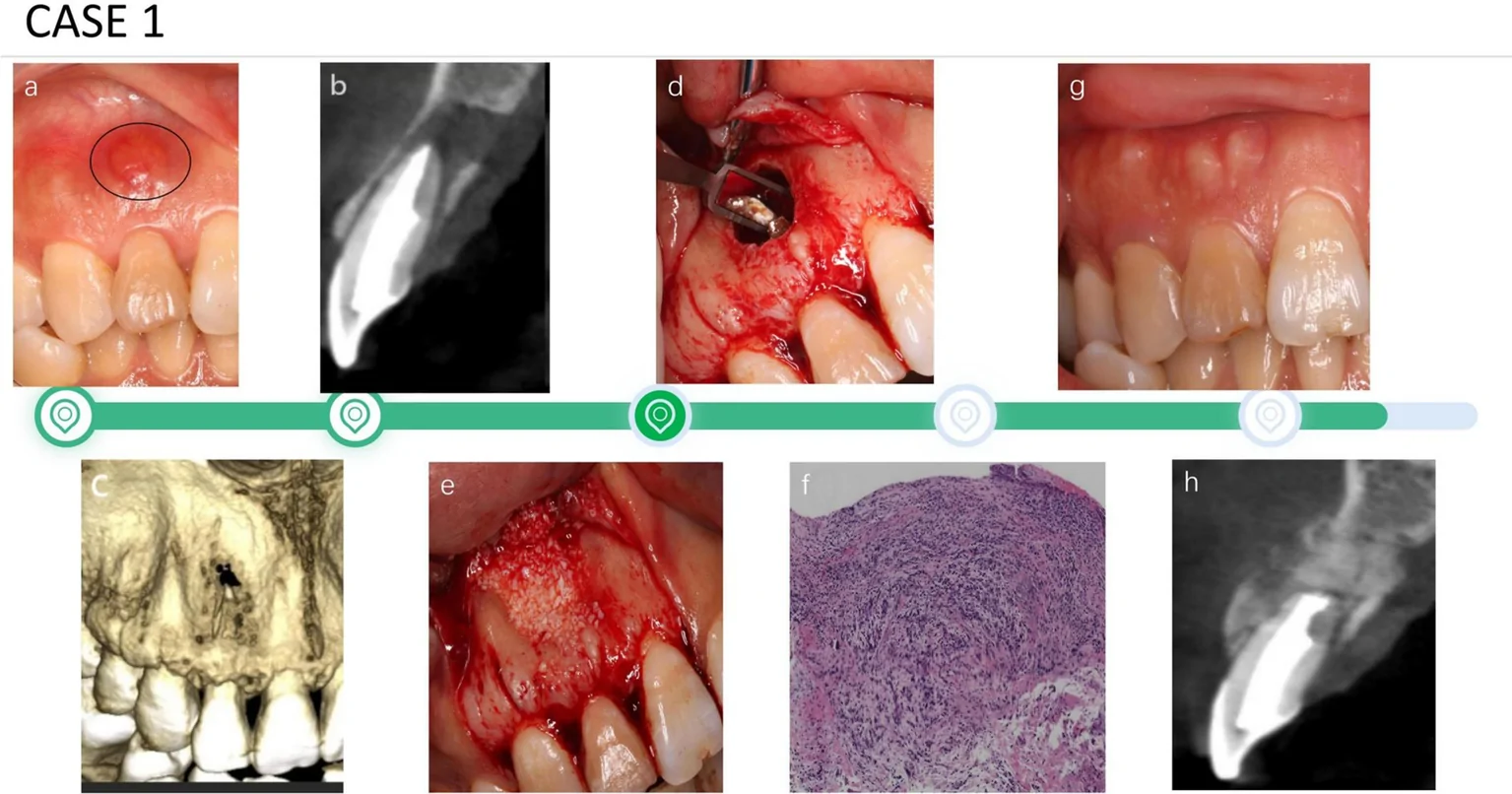
Case 1: Tooth#7 involving penetration of the labial cortical bone. **(a)** The initial intraoral photograph showing the buccal mucosa near tooth#7 is red in the circular. **(b,c)** A large apical radiolucency encompassing tooth#7 and the large defect of cortical bone in CBCT. **(d)** After flap elevation and lesion curetting, root tip resection, and sealing of the root tip with iRootBP Plus. **(e)** The apical bone defect of tooth#7 was restored with GBR. **(f)** Inflammatory cells consisting of lymphocytes, plasma cells, and histiocytes in the epithelium of the radicular cyst. **(g)** A sinus tract disappearing and the gingiva in good condition at the 12-month follow-up. **(h)** 12-month follow-up CBCT axial view slices showing complete healing.

A 28-year-old medically fit woman complained of gingival swelling and masticatory pain in the maxillary right anterior region for 3 months. Several years before visiting our department, she received RCT for tooth #7 at a local hospital and underwent repeated RCT 1 year prior due to gingival swelling. Clinical examination revealed that tooth #7 had no response to cold testing and no percussion sensitivity. The crown of tooth #7 appeared brownish-grey, with a defective incisal edge. CBCT showed that tooth #7 had a large through-and-through periapical lesion (8.5 mm × 7.5 mm × 9.0 mm), and radiopaque root filling material was extruded beyond the root apex. Accordingly, EMS and curettage for tooth #7 were performed. Root tip resection and apical sealing with iRoot BP Plus (BC, Canada) were completed, and GBR with Bio-Oss and Bio-Gide was performed for the large lesion. At the end of the procedure, the flap was repositioned and sutured. Antibiotics and ibuprofen were prescribed for the patient. Histopathological examination confirmed a periapical cyst. At the 12-month follow-up examination, the patient reported no clinical symptoms, and CBCT indicated complete healing of the lesion (see Figure 6).

#### Case 2

**Figure 7:**
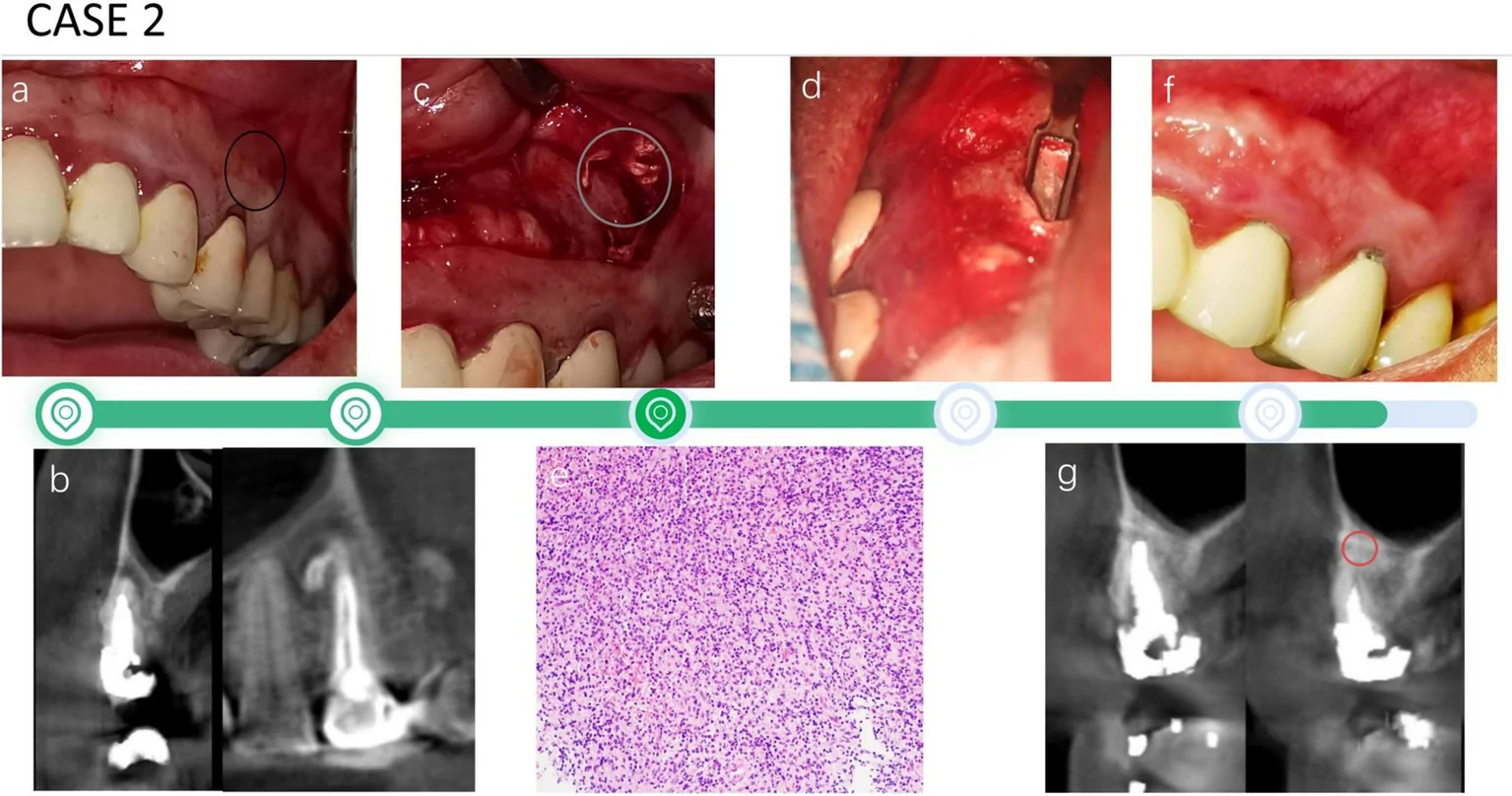
Case 2: Tooth#12 with recurrent gingival discomfort. **(a)** The buccal gingival swelling near tooth#12 is circular. **(b)** A large apical radiolucent area around the apex of tooth#12 and a tapering root filling extending to the full length of the canal. **(c)** The filling material of the root canal attaching to the periosteal surface. **(d)** Microscopic apical excision and root canal retrofilling with iRootBP Plus. **(e)** The inflammatory cell infiltration, including histiocytes, lymphocytes, and plasma cells. Additionally, numerous neutrophils, well-defined foamy macrophages, and vascular structures in the periapex tissue. **(f)** No clinical signs or symptoms in tooth#12 at the 12-month follow-up. **(g)** Complete healing; bone tissue healing area in the circle at the apical region with GBR.

A 45-year-old male was referred to us for occlusal pain in the maxillary left premolar area for several months. There was a porcelain crown restoration on tooth#12. The man reported that tooth#12 had undergone RCT 5 years ago at another hospital prior to pain, and repeated RCT for tooth#12 was performed 1 year ago because of buccal gingival pain. The examined area demonstrated that the buccal mucosa near tooth#12 was red, tooth#12 was painful to percussion, and preoperative CBCT revealed that tooth#12 had a restoration extending into the pulp chamber and a tapering root filling extending to the full length of the canal. The apical radiolucency measured 6.0 mm × 9.5 mm × 10.5 mm, and root filling material extruded past the root apex. Tooth#12 was diagnosed with periapical periodontitis. Consequently, EMS with GBR was suggested for tooth#12. The biopsy report confirmed periapical granulomas and, at the 12-month follow-up, CBCT indicated complete bone healing of tooth#12 (see Figure 7).

## Discussion

EMS has been widely performed and applied in clinical practice, successfully treating numerous teeth with refractory chronic periapical periodontitis. For an affected tooth with periapical inflammation accompanied by an extensive bone defect, GBR may be used to rebuild the periapical bone tissue. A retrospective design was used to conduct a clinical data analysis of 23 teeth from 23 patients who had undergone EMS with GBR. The overall success rate was 78.26% (18 out of 23). The success rate was largely consistent with previous literature reports (15) (69%–93%), and all cases exhibited severe lesion severity of Category F in the Kim & Kratchman classification (17) The age range was 20–70 years, with most patients aged 20–50 years. Refractory periapical periodontitis was often accompanied by extensive periapical bone defects, and young and middle-aged patients exhibited a stronger willingness to retain their affected teeth through EMS [treatment]. No significant differences in the surgical success rates were observed among age variables. This finding was consistent with most literature reports, which indicate no age-related differences in the success rate of EMS (18). There was also no statistically significant difference between female and male patients, consistent with the findings of the study (19). An analysis of patients’ income sources revealed that a higher proportion of EMS recipients had stable incomes, suggesting that economic status might influence willingness to undergo EMS surgery, although no significant difference was observed in the success rate. The investigation of the patient’s overall condition revealed that body weight and lipid profile levels did not significantly influence the success rate of EMS. The impact of demographic factors on the success rate of EMS was consistent with previous research (20). Regarding behavioral habits, statistical analysis was conducted on oral health status and smoking habits. Neither factor showed a significant impact on the EMS success rate. Truschnegg et al. (21) showed a lower success rate in smokers (33.3%) when compared to non-smoking patients (80.4%) after 10–13 years of EMS. The variable findings might be related to observations with the follow-up period. The primary occlusal parameters recorded for EMS included crowding depth, overbite/overjet, and crossbite. The incidence rates of crowding and crossbite were essentially equivalent among patients. Moreover, it had no significant impact on the association of EMS. In the overbite/overjet group, the surgical success rate was only 50.00%, whereas it was 93.33% for those without these conditions, with a statistically significant difference. The result was consistent with numerous previous studies on oral mechanics. The analysis results of Ran et al. (22) showed apical root resection resulted in a marked increase in stress distribution and tooth displacement in normal and increasing overjet with deep overbite occlusal relationships, while the stress distribution and tooth displacement increased markedly with overjets or deep overbites. Küçük Keleş and Eraslan (23) evaluated the biomechanics of maxillary first molar teeth following palatal, disto-buccal, and mesio-buccal root amputation after EMS *in vitro* environment using Reciproc R25 files and 3D Slicer software, suggesting a higher biomechanical risk than disto-buccal or mesio-buccal resections and the highest von Mises stresses in root dentin after EMS. This was a similar conclusion to Alharbi’s et al. (24) vitro study on a single mandibular premolar, and the research results demonstrated resistance to pore formation under simulated occlusal stress. The efficacy of EMS might be dependent on the integrity of the apical occlusal seal and the adaptability of the apical filling material under simulated occlusal stresses. Three cases of crown fracture occurred 1 year postoperatively, accounting for 60% of the overall failure rate in the study. The potential causes include reducing tooth root length after root amputation and diminishing the buffering capacity of the apical region, combined with the patient’s pre-existing occlusal relationship and uneven force distribution during mastication, ultimately leading to treatment failure. That might suggest that follow-up is essential for patients with overbite/overjet and regular occlusal, and crown restoration should be performed when necessary. The above conclusions are based solely on the results of a retrospective study involving a limited sample size. This might be a false positive discovery resulting from overfitting, so the conclusions require further validation through extensive cases with 5-year follow-up and RCTs.

Preoperative clinical symptom analysis revealed that gingival swelling and pain were observed in 11 cases (47.83%). Previous studies (25) found that the most common clinical symptoms in EMS were positive percussion tenderness and gingival swelling/pain, with approximately half of the affected teeth exhibiting sinus tracts and apical tenderness in oral examination. Affected teeth typically did not present with severe or acute pain (26). An appropriate treatment plan should be determined as early as possible based on the patient’s medical history, treatment history, clinical examination, and radiography (27). A minority of teeth were diagnosed with combined periodontal and pulp diseases, accompanied by periodontal pockets or mobility (28). Microorganisms establish pathological communication between the pulp and periodontal tissues through the apical, collateral root canals, or dentinal tubules, thereby mutually influencing each other and exacerbating periodontal or periapical inflammation (29). Moreover, extensive periapical lesions were combined with periodontal disease and exhibited poor prognosis (30). Some studies (31) have shown that the success rate of EMS was significantly reduced in teeth with periapical lesions accompanied by periodontal issues. However, our study showed that periodontitis was not significantly impacted by the preliminary association of EMS, which might be due to the comprehensive and meticulous evaluation of the periodontal condition of cases at perioperation and follow-up, along with the implementation of appropriate periodontal treatment measures. Therefore, both periodontal treatment and RCT should be performed simultaneously to prevent disease progression for EMS patients. It Periodontal examination is crucial during clinical follow-up. Similar findings were also revealed in our retrospective research: two cases had periodontal and pulp lesions and one case developed periodontal infection remaining uncontrolled after EMS. Another case experienced an infection of the periapical grafting, ultimately leading to EMS failure. Dental caries and periodontal diseases accounted for the highest proportions, at 52.17 and 30.43% in the initial etiological association, respectively, while a history of dental trauma represented a lower proportion. The initial etiology was not significantly influenced by the preliminary association of EMS. Particularly, the study with the highest proportion of teeth with a history of dental trauma exhibited discrepancies in findings (32). The 23 cases in this study may involve larger, more complex, or through-and-through lesions and therefore may not be fully representative of all teeth treated with EMS. Consequently, definitive conclusions require further validation through larger cohorts with 5-year follow-ups and randomized controlled trials.

Of the 23 cases, 13 had undergone RCT, six cases were re-RCT, and four cases did not receive RCT. The apical cysts of four cases were extensive in size. Considering the time constraint of patients, RCT treatment was performed concurrently with EMS during the same procedure. To ensure uniformity in the statistical evaluation of RCTs, the procedures were performed under microscopic guidance by the same senior endodontist. For the majority of completed cases, the initial RCT was performed at another institution; consequently, detailed information regarding the initial treatment status could not be obtained from medical records or radiographical data. The quality of root canal filling was typically assessed through radiographic examination. Through comparative analysis of postoperative success rates, it was found that preoperative RCT had no significant impact on outcomes. In other words, as long as a comprehensive RCT had been performed, the RCT was not statistically different in the prognosis of EMS. The RCT was crucial for EMS, as the quality of root canal filling was directly determined by persisting periapical lesions, and the findings of this study were consistent with this conclusion (33). Analysis of the apex diameter revealed no significant impact of root canal diameter on the success rate of EMS. Similarly, it was predominantly located in the anterior region (13 cases, accounting for 56.52%) (34), yet there was no notable difference in the distribution of tooth positions regarding EMS outcome. This differs slightly from the findings of the study (35), which showed early eruption of anterior teeth was coupled with delayed treatment following trauma during childhood or adolescence. This condition led to halting root development, enlarged root canals, incomplete formation of the apical foramen, and increased difficulty in RCT, which became a major contributing factor to poor prognosis in EMS (36). The potential reason for the observed discrepancies may lie in the fact that this retrospective study primarily focused on cases undergoing GBR concurrently with EMS, resulting in a limited scope of coverage. Furthermore, multiple radiographic imaging studies suggested densely filled teeth (37), and the apical filling might not be entirely airtight, producing imaging discrepancies. Unfilled collateral root canals might also exist, and intraoperative root and apical cross-sectional staining examinations still detected root canal leakage, indicating that endodontic infections were spread from the apical foramen or collateral canals to periapical tissues, leading to persistent periapical infection (38). Intraoperative staining could differentiate certain collateral canals from root canal leakage, and apical resection was combined with inverting filling; all these could improve the success rate.

Through retrospective radiographical imaging analysis using Mimics software, the periapical lesion volume was extensive in our study (volume > 1,000 mm^3^, accounted for 69.57%), yet the result showed no significant impact of lesion volume on the success rate of EMS treatment. A larger periapical lesion volume was associated with more severe periapical bone destruction. Additionally, the study assessed cortical bone destruction and its influence on EMS treatment success rates: 65.23% of cases were through-and-through lesions, while 34.77% involved the unilateral cortical bone being defective in the palatal or buccal cortical plates. Extensive periosteal bone destruction was observed, although this did not significantly affect the success rate of EMS. Similarly, the GVs of periapical lesions across all lesion areas were analyzed using Mimics. Patients with GVs > 800 accounted for 56.52%, and all cases were successful in this group. In contrast, the success rate was only 33.33% in patients with GVs < 500, indicating a significant impact of GVs on EMS outcomes (P: 0.023; OR: 0.091; 95% CI: 0.011–0.717). That might be attributed to GVs reflecting periapical bone health to some extent, which were influenced by GBR in EMS. However, due to the limited proportion of cases included in the study, more cases with 5-year follow-up observation are required to confirm the result. Alveolar bone condition was critical for GBR (39). These findings align with previous studies demonstrating successful GBR grafting (40). The use of a collagen membrane depends on the presence of a sinus and without a collagen membrane in the absence of a sinus. Due to the integrity of the periosteum and the stability of the bone grafting space, some cases in this study without a collagen membrane in the absence of the sinus still achieved complete healing at the apical bone graft site, which was similar to that reported by Dudeck et al. (41). The feasibility of the approach required further validation through extensive case series and long-term follow-up. Additionally, our retrospective study utilized Mimics with 3D reconstruction at 12-month follow-up, allowing more intuitive visualization of periapical bone tissue reconstruction and enhancing the assessment of EMS with GBR outcome. However, the efficacy of combined grafting on EMS remained controversial (42). Previous studies (43) suggest that combined grafting promoted periapical tissue regeneration, particularly in penetrating or extensive lesions. Further radiographical imaging revealed that it might accelerate surrounding bone healing. However, as this was a retrospective study with a limited sample size, a randomized controlled trial would provide greater rigor to exclude confounding factors. The healing status and the integration of bone with bone graft materials requires further validation through histopathological and animal experiments.

To analyze the impact of periapical lesion tissues on the association of EMS, HE staining was performed on all cases, followed by histological classification. Of the cases, 16 (69.57%) were diagnosed as radicular cysts, while seven (30.43%) were periapical granuloma. The periapical granuloma group exhibited highly developed granulation tissue and prominent vascular structures, along with infiltration of lymphocytes, plasma cells, and numerous inflammatory cells; some cases also showed fibroblast and partial vascular distribution with less inflammatory cell infiltration. In contrast, periapical cyst tissues displayed epithelial pinnacles with chronic inflammatory cell infiltration, intercellular edema, cholesterol crystal deposition, and collagen fibers. These histological differences did not show a statistically significant correlation with EMS success rate, a finding consistent with existing reports (41). This may be because the periapical lesion tissue was not completely removed in other studies (44). In our study, the periapical tissue had been eliminated, so the periapical lesion tissue did not significantly affect the success rate of EMS with GBR. Some studies (45) also showed that incomplete removal of periapical lesion tissue might relate to the failure to achieve complete clearance in extensive periapical lesions, leading to cumulative cortical bone resorption or gingival fistulas, which result in a minimal influence of the periapical lesion tissue structure on the outcome.

## Conclusion

This study conducted a retrospective analysis of the clinical data from 23 cases involving 23 teeth in EMS with GBR. Mimics software was utilized to assess periapical lesion morphology and preoperative and postoperative CBCT data, as well as to evaluate the potential associations between preoperative dental status, periapical lesion characteristics, demographic distribution, malocclusion variables, and surgical success rates. Univariate analysis indicated that a statistical association between the gray values of periapical lesions (*p* = 0.023; OR = 0.091; 95% CI: 0.011–0.717) and deep overbite/overjet parameters (*p* = 0.035; OR = 0.071; 95% CI: 0.006–0.834) was observed in this small sample. However, the positive findings derived from association analysis in this study carry a risk of overfitting due to the limited sample size. Therefore, validity and stability urgently require validation in future large-scale prospective studies, which are warranted to confirm these associations. 3D reconstruction in Mimics effectively visualized periapical radiographic changes, enhancing the intuitiveness and reliability of outcome analysis; its added value over standard CBCT assessment remains unclear. No significant direct correlation was observed between histopathological morphologies and EMS with GBR. As these conclusions are derived solely from a small-scale retrospective analysis, definitive conclusions regarding these associations require validation through larger prospective studies with extended follow-up and randomized controlled trials.

## Data Availability

The datasets presented in this study can be found in online repositories. The names of the repository/repositories and accession number(s) can be found in the article/supplementary material.
